# Direct Application of Coffee Pulp Vermicompost Produced from Epigeic Earthworms and Its Residual Effect on Vegetative and Reproductive Growth of Hot Pepper (*Capsicum annuum* L.)

**DOI:** 10.1155/2023/7366925

**Published:** 2023-12-19

**Authors:** Yohannes Zergaw, Temesgen Kebede, Dargie Tsegay Berhe

**Affiliations:** Dilla University, College of Agriculture and Natural Resources, Dilla, Ethiopia

## Abstract

In areas where coffee is growing, there is a huge potential to get coffee pulp, which produces a bad odor and air and water pollution. On the other hand, the farming practice in the study area is still traditional and highly dependent on artificial fertilizers. Therefore, this study aimed to evaluate the direct and residual effects of vermicompost on the vegetative and reproductive growth of hot pepper (*Capsicum annuum* L.). Factorial research was conducted in a randomized complete block design, where coffee pulp, animal waste, and *Eisenia fetida* and *Dendrobaena veneta* earthworms were used to produce coffee pulp vermicompost. The first factor was three types of vermicompost: coffee pulp *Eisenia fetida* vermicompost (CEV), coffee pulp *Dendrobaena veneta* vermicompost (CDV), and a combination of coffee pulp *Eisenia fetida* and *Dendrobaena veneta* vermicompost (CEV_CDV), and the second factor was three application rates (5 t/ha, 10 t/ha, and 15 t/ha) and a control treatment with three replications. Reproductive and vegetative growth parameters (number of leaves, number of fruits, single fruit length, total biomass weight, days to 50% flowering, and yield per hectare) were collected in two consecutive cropping seasons. The collected data were analyzed using R Statistical software. The results recorded in the direct application of 15 t/ha CEV were profoundly higher (*P* < 0.05), followed by the combined effects of both worms with an application rate of 15 t/ha. Generally, the three-way interaction effect of a media type, the application effect, and the application rate interaction effect were highly significant (*P* < 0.05) for the number of leaves per plant, the number of fruits per plant, and yield per hectare. The media type and the application rate interaction effect showed a significant (*P* < 0.05) difference in days to 50% flowering, fruit length, and fresh biomass weight. Likewise, the combined effect of the media type and application was statistically (*P* < 0.01) different in biomass weight and fruit length. In addition to reducing water and air pollution, utilizing coffee pulp vermicompost enhances soil fertility and hot pepper (*Capsicum annuum* L.) productivity.

## 1. Introduction

The second most important vegetable crop in the world following tomato is hot pepper (*Capsicum annuum* L.), which belongs to the Solanaceae family [1]. It has essential vitamin and mineral content, spice flavouring, and colour for food, as well as natural pest control potential [2]. Hot pepper (*Capsicum annuum* L.) is growing in different parts of Ethiopia, mainly in Amhara, Oromia, and Southern Nations, Nationalities, and Peoples Regional State (SNNPRS). The crop contributes to an increase in overall national production of peppers of about 43% in SNNPRS. In most parts of the country, it is consumed as vegetables, spices, or condiments as a part of daily consumable foods [3]. On the top of its monocrop nature, the crop grows with other cereals and legumes in rotation and can even grow in dry seasons with the help of irrigation water supply in lowland areas of the country where the climatic condition is conducive [4].

In Ethiopia, poor crop productivity is mainly associated with poor soil fertility and loss of macronutrients via soil erosion and other natural and anthropogenic factors in different parts of the country [5]. Moreover, low soil fertility and poor quality of seeds [6], temperature, soil, humidity, light intensity, and moisture [7], and vegetative growth performance and yield of hot pepper (*Capsicum annuum* L.) are highly reduced [1, 3]. In addition, the country's intensive soil cultivation, improper use of inorganic fertilizers, and poor soil fertility management practices have reduced soil fertility, primarily through the depletion of soil nutrients and the reduction of the yield of crops, particularly hot pepper (*Capsicum annuum* L.) [8]. Soil nutrient management and selection of improved crop varieties are vital for pepper productivity which helps to increase producers' income so as to boost their livelihood [9]. Thus, the application of organic amendments such as vermicomposting, decayed leaves, compost, sawdust, or animal manure is very important to improve the soil fertility and yield of hot pepper (*Capsicum annuum* L.) [10].

Vermicomposting (composting with the use of worms) is a new organic fertilizer utilization approach using the quick recycling of organic materials into nutrient-rich compost with the help of earthworms such as *Eisenia fetida* and *Dendrobaena veneta* under aerobic conditions. Vermicomposts are rich in beneficial soil microorganisms which help in soil regeneration and fertility improvement [11]. Moreover, epigeic earthworms such as *Eisenia* fetida are used for vermicomposting, which have the capacity to improve soil organic matter and can even transform organic residues both physically and chemically [12]. Vermicomposts are prepared from homestead wastes, agricultural practices such as coffee production and processing, poultry, cow dung, and sawmill and even from tannery wastes.

Numerous studies have been conducted to evaluate the ameliorating effect of vermicompost on soil fertility, growth, and yield of crops. Among different researchers, Gebresilassie and Israel [7] and Anna et al. [13] reported that the application of vermicompost is well known for its effects on plant nourishment and ease of uptake, which results in reduced requirements for (or the elimination of) chemical pesticides and inorganic fertilizers [12]. Similarly, its potential to increase agronomic yield is greater than crops amended with artificial fertilizers and cow dung [14, 15].

There is a huge potential to produce vermicompost by using organic wastes released from local industries such as coffee processing industries in southern parts of Ethiopia and coffee-growing areas in particular, but there is a controversy on the rate of application of these organic fertilizers prepared from different organic materials, which is very much important to increase its efficiency for crop production in the direct application and residual effect [16]. Afroja et al. [17], Kassa et al. [11], and Rasool et al. [18] indicated that soils fertilized with vermicomposting have the potential to increase residual zinc, manganese, organic carbon, and porosity and decrease pH. Besides the high direct production effect of vermicompost applied, its residual effect on crop production is very high. Soil fertilized with vermicomposting shows higher residual nitrogen, organic carbon, and phosphorous fertilizer than inorganic fertilizers [14], but the direct and residual effects of vermicompost are determined by the vermicompost quality, application rate, and nature of the crop to be grown [15]. Thus, the purpose of this study was to determine the optimum rate of coffee pulp vermicompost organic fertilizer prepared by using two different epigeic earthworms in direct application on hot pepper (*Capsicum annuum* L.) and to evaluate its residual effect.

## 2. Materials and Methods

### 2.1. Description of the Study Area

The field experiments were carried out in *Tokicha Kebele*, Wonago District, Gedeo Zone, Ethiopia, in the 2022 cropping season under irrigation. The area is located at 7°20′ 05″ north latitude and 36° 17′ 40″ east longitude with an altitude of 1700 m.a.s.l. and is located 420 m south of Addis Ababa. The district is characterized by 980–1700 mm annual rainfall and 12–25°C temperature. Vegetables, coffee, enset, and different horticultural crops are highly grown in the area, and there are a number of local coffee processing industries causing water and air pollution in the surrounding environment. There is also immense animal manure waste available due to potential animal production in the area.

### 2.2. Collection of Vermicompost Substrates and Production Procedures

Coffee pulp was collected from wet coffee processing industries of Wonago District, and cow manure was collected from Dilla University animal farm areas. Furthermore, both *Eisenia fetida* and *Dendrobaena veneta* earthworms were collected from the Dilla University vermicompost production project site. The selection of these organic wastes was based on the large accumulation and great potential in the study area. Vermicomposts with a mixture of 75% coffee pulp and 25% cow dung [19] were prepared in 2 m × 1 m × 1 m boxes and cocomposted for thirty days, to break down the substrates into smaller parts that are more easily appetizing to vermin worms. Both *Eisenia fetida* and *Dendrobaena veneta* adult earthworm species were introduced into the vermicomposting boxes after cocomposting mixed materials. Vermicompost was produced and harvested after thirty days by following the recommended procedures and appropriate media [19].

### 2.3. Experimental Design and Treatments

The field experiment was conducted in a randomized complete block design with a factorial experiment in two consecutive seasons on irrigable farmer's farmland. The first factor was three types of vermicompost: coffee pulp *Eisenia fetida* vermicompost (CEV), coffee pulp *Dendrobaena veneta* vermicompost (CDV), and a combination of coffee pulp *Eisenia fetida* and *Dendrobaena veneta* vermicompost (CEV_CDV). The second factor was three application rates (5, 10, and 15 t·ha^−1^) and a control treatment with three replications. There were nine treatment combinations and one control treatment. In total, there were ten treatments with three-plot replications. The residual effects of the treatments were also evaluated as the third factor in the field experiment. Vermicompost was applied, well mixed, and watered 15 days before transplanting seedlings to the research plots.

### 2.4. Seedling Preparation and Transplanting

20 grams of local landrace hot pepper (*Capsicum annuum* L.) seed was collected from vegetable producer cooperative farmers. The seed was drilled on three beds with sizes of 1 m × 5 m at a spacing of 10 cm along rows and covered with dry grass. Management of seedlings such as watering, weeding, thinning, and protection was practiced in the nursery site and they were transplanted at the 4 true leaf stage (15cm tall) usually six weeks after sowing [20]. Shades were also removed, and hardening was practiced by reducing the amount of water supplied. Then, the experiment field was ploughed and leveled, and thirty plots were prepared with an area of 2.88 m^2^. Moreover, the vermicomposts were applied and mixed with soils at a depth of 20 cm in each plot two weeks before transplanting seedlings. Finally, strong and healthy hot pepper (*Capsicum annuum* L.) seedlings were properly uprooted and planted in well-prepared plots with a spacing of 30 cm between plants and 40 cm between rows. Management of planted seedlings was also practiced until harvesting hot pepper (*Capsicum annuum* L.) yield. This procedure of raising and transplanting seedlings was also carried out in the second experimental season.

### 2.5. Data Collection Methods

#### 2.5.1. Agronomic Data Collection

For hot pepper (*Capsicum annuum* L.), in each treatment, twelve plants were randomly selected and tagged for various agronomic parameters. Days to 50% flowering (DTF) was determined by the number of days from emergence until 50% of plants per plot attained first flowering. The number of fruits per plant in each treatment was harvested by picking and counted to calculate the average fruits/plant. Fruit length was measured using a meter scale. The fresh weight of the fruit (g) was measured just after harvesting with the help of an electronic weighing machine. Fruit yield was determined by adding the total fruit weight over all the pickings from each plot and converted on a hectare basis by using the following formula [21]. These agronomic data parameters were repeated in the second growing season.

(1) $$\begin{array}{c} \text{Yield }\left(\text{tones per ha}\right)=\frac{\left(\text{Subplot yield }\left(\text{ton}\right)\times 10000{\;m}^{2}\right)}{\left(\text{Subplot area }\left(m^{2}\right)\times 1000\right.}. \end{array}$$

#### 2.5.2. Soil Data Collection Method

Soil organic carbon was determined using the wet oxidation method [22]. The total N of the soil and compost was determined through the micro-Kjeldahl method as described by Jackson [23]. Determination of available phosphorous and available potassium was carried out by the Olsen method using sodium bicarbonate (0.5M NaHCO_3_) as the extraction solution [24]. Exchangeable bases (Ca, Mg, K, and Na) in the soil were estimated by the ammonium acetate (1M NH_4_OAc at pH 7) extraction method [25]. In this procedure, the soil samples were extracted with an excess of NH_4_OAc solution and Ca and Mg in the extracts were determined by using an atomic absorption spectrophotometer [26], while a flame photometer [27] was used to determine the contents of exchangeable K and Na as described by Rowell [28]. Nutrient compositions (organic carbon, total nitrogen, C : N ratio, phosphorous, and potassium) of coffee pulp vermicompost prepared from two different epigeic earthworms were also determined.

### 2.6. Statistical Analysis

The data collected were subjected to the analysis of variance (ANOVA), and the analyses were carried out using the R Statistical software (version 4.1.3, 2022); any differences were compared at *P* < 0.05 among the investigated parameters using Tukey's comparison test. The significant differences between treatment means were calculated with the help of Fisher's range test at a 5% significance level (*P* < 0.05).

## 3. Results and Discussion

### 3.1. Properties of Organic Amendments

**Figure 1:**
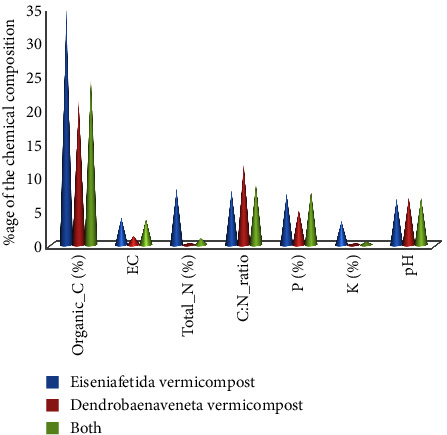
Physicochemical compositions of coffee pulp vermicompost.

The properties of organic amended soil used for the study are presented in Figure 1. The study result revealed that the highest application rate of CEV was higher in its organic carbon, electrical conductivity (EC), total nitrogen, and available phosphorous and available potassium (Figure 1). A higher C : N ratio and soil pH were recorded from the maximum application rate of CDV, but the highest *P* value was recorded from the highest application rate of CEV_CDV.

Physicochemical composition of coffee pulp vermicompost may be varied due to the nature of worms utilized and their capacity to alter the organic materials added to the vermicompost. Berhe et al. [21] and Kulandaivelu and Kurian [29] reported the highest organic carbon, total nitrogen, available phosphorous, exchangeable potassium, calcium, and magnesium, and pH values in CEV compared to CDV. This result was comparable and similar to [30–33].

### 3.2. Number of Leaves per Plant

The three-way factorial effect of the media type, application effect, and application rate was notably varied (*P* < 0.01) in the number of leaves per plant (Table 1). The maximum number of leaves per plant was registered from the direct application of 15 t/ha CEV (499.33 ± 50.64) followed by the direct application of 15 t/ha CEV_CDV (410.01 ± 18.73), while the minimum values were recorded in the residual effect of the control treatment (20.04 ± 2.65) and direct application of the control (28.67 ± 2.52). The number of leaves per plant in the direct application of 15 t/ha CEV was higher by 95.99% than the residual effect of the control. In both application effects, numbers of leaves were significantly different among the different rates of the same media type. Residual effects of 15 t/ha CEV application were even significantly higher (361.33 ± 14.22) in the number of leaves per plant than in 10 t/ha CDV which was used in the direct application (306.68 ± 17.90). This result indicated that when the rate of vermicompost application was increased in all treatments, the number of leaves was also increased in direct application and also for its residual effect. Likewise, the crop supplied with CEV has produced more number of leaves followed by the combined application of CEV_CDV.

The number of leaves of the crop amended by 15 t/ha CEV has shown a higher number than the other treatments in both direct application and residual effects. The highest number of leaves observed in 15 t/ha CEV might be due to the nature of worms to produce important enzymes and growth hormones and the increased amount of total fresh biomass of the crop. A similar finding by Esperanza et al. [5] indicated that the application of vermicomposts increased the number of leaves of Amashito pepper in advance of other organic fertilizers such as cow dung and cocoa husk. The highest leaf number production was recorded in strawberry, garlic, and tomato when the amount of vermicompost applied to the crop was increased [34]. In addition, Bewuket [35] reported that the application rate of vermicompost increased the amount of vermicompost from 10 t/ha to 15 t/ha which has a positive effect on the number of leaves per plant in the case of tomato and pepper. In a similar manner, the findings of Dalorima et al. [31] and Alemu et al. [33] showed that a larger number of leaves and an increased leaf area were recorded from watermelon crop. This result was also supported by the findings of Berhe et al. [21] and Ali et al. [36] who reported that a larger number of leaves were recorded from pepper supplied with the highest application rate of CEV in both direct application and residual effect cases followed by the maximum application of CEV_CDV.

### 3.3. Number of Fruits per Plant

The three-way interaction effects of the treatments showed a significant difference (*P* < 0.05) in the number of fruits produced per plant. The direct application of 15 t/ha CEV showed the highest number of fruits per plant (222.33 ± 9.29) followed by the application of 15 t/ha CEV_CDV (189 ± 12.50) and 10 t/ha CEV (184.32 ± 4.04) (Table 2), while the least number of fruits per plant was recorded from the residual effect of the control (24.33 ± 3.51). The direct application of 15 t/ha CEV produced 89.06% higher number of fruits per plant than the residual effects of the control. There were an increased number of fruits per plant when the application rate was increased from 0 to 15 t/ha of CEV. The direct application of CEV produced a higher number of fruits per plant when applied directly followed by the combined application of CEV_CDV.

The highest fruit number was obtained from the crop produced using CEV with an application rate of 15 t/ha which might be due to the ability of vermicompost to improve the root rhizosphere so as to improve crop growth and to produce more fruits. Moreover, the application rate of 15 t/ha CEV can improve soil organic matter and enhance the availability of macronutrients as well as micronutrients in the crop root zone. In line with this result, Esperanza et al. [5] found that the highest application rate of vermicompost increased the number of fruits of the Amashito crop. Hazrat et al. [37] also revealed that the highest application rate of vermicompost even produced the maximum number of fruits of chilli. This finding was also in agreement with the finding of Berhe et al. [21] who mentioned that maximum fruit numbers were recorded from the plant supplied with the maximum application rate of CEV followed by the highest application of CEV_CDV.

### 3.4. Yield per Hectare

The combined effects of the media type, application rate, and application effect on crop yield were statistically (*P* < 0.01) different (Table 3). The direct application of 15 t/ha CEV produced the maximum yield (26.21 + 1.19) followed by 15 t/ha CEV_CDV (21.56 ± 2.57) and 10 t/ha (20.34 ± 1.77). Minimum crop yield per plant was recorded from the residual effects of the control treatment. Crop yield from the direct application of 15 t/ha CEV was 97.86% greater than crop yield from the residual effect of the control treatment. Similar to the above results, a higher yield was recorded when the application rate was increased and applied directly to the crop.

Due to the availability of essential nutrients in the optimum amount in the crop root zone and improving the activities of soil microorganisms, a significantly higher yield was recorded in the application of 15 t/ha CEV than in the application of 15 t/ha CDV. In addition, the application of 15 t/ha CDV creates a conducive environment in the crop rhizosphere by maintaining moisture and providing food for the microbes, producing growth hormones for the crop and increasing the crop yield. The current result is in agreement with the finding of Bewuket [35] who reported that the application of a 15 t/ha vermicompost for okra crop increased its yield by 70–100%, 75% for banana and 50% for sunflower than the recommended amount of inorganic fertilizer. Geremew [34] revealed that a soil amended with an increased rate of vermicompost could be rich in soil-available micro- and macronutrients and can be stored for a long time, which increases the crop yield.

### 3.5. Days to 50% Flowering

The interaction effect of days to 50% flowering was significantly different (*P* < 0.001) among the rates of treatments (Table 4). The shortest days of 50% flowering was recorded in 15 t/ha CEV (44.00 ± 7.72), followed by 15 t/ha CEV_CDV (50.33 ± 3.44) and 10 t/ha CEV (53.50 ± 4.04), while the longest days to 50% flowering was observed in the control (85.67 ± 5.05).

The shortest days to 50% flowering was registered in the direct application of 15 t/ha CEV which might be due to the availability of soil organic materials and improve the vegetative and reproductive growth of the crop. In addition, the reduced flowering period in the direct application of 15 t/ha CEV could be associated with fast plant growth and energy to produce flowers and even seeds or fruits. In the current study, days to 50% flowering was very fast and short for the crop amended with the maximum application of 15 t/ha. The report of Berhe et al. [21] and Olowoake et al. [14] indicated that the direct application of 15 t/ha CEV reduced the number of days to 50% flowering of hot pepper (*Capsicum annuum* L.).

A similar result was found by Dalorima et al. [31] that application of vermicompost with 15 t/ha has shown the shortest days to 50% flowering of watermelon. Accordingly, Mengistu et al. [38] stated that the tomato crop amended with 15 t/ha *Eisenia fetida* vermicompost reduced days to 50% compared with the other treatments. The reduced number of days to 50% flowering of crops supplied with 15 t/ha vermicompost can be associated with fast activities of soil micro-organism production of growth hormones and release of beneficial enzymes which can help to improve growth and early blooming from the vermicompost.

### 3.6. Fruit Length

The interaction effect of the media type and the application rate on the mean fruit length showed a significant difference (*P* < 0.05). The maximum fruit length was recorded in the direct application of 15 t/ha CEV (4.75 ± 0.49), followed by 15 t/ha CEV_CDV (4.35 ± 0.34), while the minimum fruit length value was recorded in the control (2.50 ± 0.34) (Table 4).

The maximum fruit length was recorded from 15 t/ha CEV which may be due to the availability of growth hormones, improved organic materials in the crop rhizosphere, and important enzymes resulted from the vermicompost. This result is in agreement with Hazrat et al. [37] who found a maximum fruit length from chilli crops supplied with vermicompost which emphasized that the application of vermicompost increases the availability of macronutrients in the soil which help to produce long fruits.

Similarly, Olowoake et al. [14] revealed that the application rate of vermicompost positively affects fruit length of Cucumber crop. This result was also supported by the findings of Berhe et al. [21] who reported that longer fruit length was recorded from the direct application of 15 t/ha *Eisenia fetida* vermicompost followed by 15 t/ha combination of *Eisenia fetida* and *Dendrobaena veneta* vermicompost, which might be due to the ability of the worms to produce hormones for crop growth so as to increase the fruit length of pepper.

### 3.7. Biomass Weight

The interaction effect of the media type and the application rate on the mean biomass weight was statistically (*P* < 0.001) significant. The highest total biomass weight was recorded in 15 t/ha CEV (266.83 ± 49.77), followed by 15 t/ha CEV_CDV (253.33 ± 45.21) and 10 t/ha CEV (244.00 ± 42.05), while the least total biomass weight was recorded in the control (81.01 ± 13.99) (Table 4). The highest biomass weight in 15 t/ha CEV could be due to the optimum availability and gradual release of micronutrients and macronutrients to plant roots which can be related to the availability of plant growth hormones and other important soil nutrients for the plant growth to increase the total biomass weight of treatment plots.

The findings of Nurhidayati et al. [16] and Berhe et al. [21] revealed that a crop amended with the direct application of 10–15 t/ha CEV produced a larger total biomass weight than the other treatment rates. Moreover, the report by Walkley and Black [22], Mengistu et al. [38], and Yatoo et al. [39] indicated that the increased rate of application of vermicompost positively influences the mean total biomass weight of vegetable crops. The reason might be due to the number and availability of important soil microorganisms and the release of enzymes which promote plant fresh biomass growth. Berhe et al. [21] revealed that the maximum biomass weight observed from the direct application of 15 t/ha CEV was greater than that of the other media types.

## 4. Conclusion

This study aimed to assess the effect of coffee pulp vermicompost on the agronomic and soil properties of hot pepper (*Capsicum annuum* L.). In the field experiment, hot peppers (*Capsicum annuum* L.) responded positively to the application of organic amendments; the highest application rate of coffee pulp from *Eisenia fetida* vermicompost (CEV) significantly increased plant growth parameters (number of leaves, number of fruits, fruit length, and biomass weight) and yield compared to other treatments. Moreover, the highest values of soil organic carbon, total nitrogen, available phosphorous, exchangeable potassium, calcium, and magnesium, and pH were recorded in soil treated with the maximum application of CEV. The conclusion to be drawn suggests that an organic amendment of 15 t/ha of CEV applied to the soil could produce a long-term beneficial effect on the soil nutrients and contribute to improving plant parameters and increasing the yield of hot pepper (*Capsicum annuum* L.). However, the response of different vegetable crops to different application rates of CEV utilized in the experiment needs further study.

## Figures and Tables

**Table 1:** The three-way interaction effect of the media type, application rate, and application effect on the number of leaves per plant of hot pepper *Capsicum annuum* L.).

Application effects	Media type	Number of leaves per plant		
		Application rate		
		5	10	15
Direct	CEV	271.31 + 2.52^g^	327.00 ± 4.36^d^	499.33 + 50.64^a^
	CDV	242.66 + 2.52^hi^	276.34 ± 7.23^fg^	306.68 + 17.90^de^
	CEV_CDV	258.33 + 3.06^gh^	297.30 ± 3.05^ef^	410.01 + 18.73^b^
	Control	28.67 + 2.52^l^		

Residual	CEV	208.03 + 10.44^jk^	315.00 ± 6.24^de^	361.33 + 14.22^c^
	CDV	200.00 + 2.02^k^	217.33 ± 4.04^jk^	242.66 + 10.26^hi^
	CEV_CDV	205.65 + 5.69^k^	231.66 ± 21.78^ij^	319.03 + 13.00^de^
	Control	20.04 + 2.65^l^		

Mean	261.92			
LSD	24.76			
CV	5.73			
*P* value	^*∗∗*^			

CEV = coffee pulp *Eisenia fetida* vermicompost, CDV = coffee pulp *Dendrobaena veneta* vermicompost, CEV_CDV = a combination of *Eisenia fetida* vermicompost and *Dendrobaena veneta* vermicompost, LSD = least significant difference, and CV = coefficient of variation. Similar letters under the same column are not significantly different, ^*∗∗*^*P* < 0.01.

**Table 2:** The three-way combination effect of the media type, application rate, and application effect on the number of fruits per plant of hot pepper (*Capsicum annuum* L.).

Application effect	Media type	Number of fruits per plant		
		Application rate		
		5	10	15
Direct	CEV	143.10 ± 4.58^d^	184.32 ± 4.04^b^	222.33 ± 9.29^a^
	CDV	128.00 ± 2.65^efg^	158.30 ± 4.04^c^	164.67 ± 11.37^c^
	CEV_CDV	135.05 ± 2.02^de^	163.29 ± 6.51^c^	189.34 ± 12.50^b^
	Control	32.67 ± 4.73^k^		

Residual	CEV	113.04 ± 7.55^hi^	139.00 ± 4.58^de^	158.01 ± 10.54^c^
	CDV	101.68 ± 5.03^j^	120.02 ± 8.89^gh^	133.66 ± 6.69^def^
	CEV_CDV	106.01 ± 3.61^ij^	123.00 ± 5.57^fgh^	144.68 ± 6.11^d^
	Control	24.33 ± 3.51^k^		

Mean	134.22			
LSD	11.27			
CV	5.09			
*P* value	^*∗*^			

CEV = coffee pulp *Eisenia fetida* vermicompost, CDV = coffee pulp *Dendrobaena veneta* vermicompost, and CEV_CDV = a combination of coffee pulp *Eisenia fetida* and *Dendrobaena veneta* vermicompost. Means within a column followed by the same letter(s) are not significantly different at the 5% LSD test.

**Table 3:** The three-way interaction effect of the media type, application rate, and application effect on the total yield of hot pepper (*Capsicum annuum* L.).

Application effect	Media type	Yield		
		Application rate		
		5	10	15
Direct	CEV	10.30 ± 0.63^g^	20.34 ± 1.77^b^	26.21 + 1.19^a^
	CDV	3.23 ± 0.74^ij^	10.47 ± 0.73^g^	16.67 ± 1.19^c^
	CEV_CDV	4.37 + 0.94^i^	13.84 ± 1.21^ef^	21.56 ± 2.57^b^
	Control	0.70 ± 0.13^k^		

Residual	CEV	7.49 ± 0.89^h^	13.35 ± 1.18^ef^	15.59 ± 0.68^cd^
	VCD	2.16 ± 0.77^jk^	9.11 ± 0.28g^h^	12.37 ± 0.59^f^
	CEV_CDV	2.48 ± 0.69^j^	9.22 ± 0.29^g^	14.44 ± 0.88^de^
	Control	0.56 ± 0.07^k^		

Mean	10.72			
LSD	1.71			
CV	9.66			
*P* value	^*∗∗*^			

CEV = coffee pulp *Eisenia fetida* vermicompost, CDV = coffee pulp *Dendrobaena veneta* vermicompost, and CEV_CDV = a combination of coffee pulp *Eisenia fetida* and *Dendrobaena veneta* vermicompost. Means within a column followed by the same letter(s) are not significantly different at the 1% LSD test.

**Table 4:** Interaction effect of the media type and application rate on days to 50% flowering, fruit length, and biomass weight of the crop.

Media type	Application rate	Days to 50% flowering	Fruit length (cm)	Biomass weight (g)
Control	0	85.67 ± 5.05^a^	2.50 ± 0.34^h^	81.00 ± 13.99^g^
CEV	5	71.67 ± 3.56^c^	3.87 ± 0.17^ef^	196.67 ± 41.71^e^
CDV	5	80.67 ± 3.98^b^	3.40 ± 0.32^g^	171.00 ± 39.45^f^
CEV_CDV	5	77.67 ± 6.92^b^	3.67 ± 0.16^fg^	177.00 ± 44.71^f^
CEV	10	53.50 ± 4.04^f^	4.27 ± 0.35^bc^	244.00 ± 42.05^c^
CDV	10	67.33 ± 5.01^d^	3.99 ± 0.16^de^	199.83 ± 37.83^e^
CEV_CDV	10	60.83 ± 4.88^e^	4.07 ± 0.19^cde^	211.83 ± 40.13^d^
CEV	15	44.00 ± 7.72^g^	4.75 ± 0.49^a^	266.83 ± 49.77^a^
CDV	15	57.67 ± 4.32^e^	4.20 ± 0.30^bcd^	218.50 ± 42.23^d^
CEV_CDV	15	50.33 ± 3.44^f^	4.35 ± 0.34^b^	253.33 ± 45.21^b^

Mean		64.93	3.91	202
LSD		3.77	0.28	7.41
CV		4.97	6.07	3.15
*P* value		^*∗∗∗*^	^*∗*^	^*∗∗∗*^

CEV = coffee pulp *Eisenia fetida* vermicompost, CDV = coffee pulp *Dendrobaena veneta* vermicompost, and CEV_CDV = a combination of coffee pulp *Eisenia fetida* and *Dendrobaena veneta* vermicompost. Means within a column followed by the same letter(s) are not significantly different at the 0.1%, 5% LSD test.

## Data Availability

The data that support the findings of this study are available from the corresponding author upon request.
